# Effects of Sodium Ferulate on Cardiac Hypertrophy Are *via* the CaSR-Mediated Signaling Pathway

**DOI:** 10.3389/fphar.2021.674570

**Published:** 2021-10-06

**Authors:** Panpan Chen, Zhaoqin Wen, Wanlan Shi, Zhongli Li, Xiaoyan Chen, Yang Gao, Shangfu Xu, Qihai Gong, Jiang Deng

**Affiliations:** ^1^ Key Laboratory of Basic Pharmacology of Ministry of Education and Joint International Research Laboratory of Ethnomedicine of Ministry of Education, Zunyi Medical University, Zunyi, China; ^2^ Key Laboratory of Basic Pharmacology of Guizhou Province, Zunyi Medical University, Zunyi, China; ^3^ Department of Pharmacology, School of Pharmacy, Zunyi Medical University, Zunyi, China; ^4^ Department of Pathophysiology, Zunyi Medical University, Zunyi, China

**Keywords:** spontaneously hypertensive rats, cardiac hypertrophy, calcium-sensing receptor, sodium ferulate (SF), hypertension

## Abstract

As a common complication of many cardiovascular diseases, cardiac hypertrophy is characterized by increased cardiac cell volume, reorganization of the cytoskeleton, and the reactivation of fetal genes such as cardiac natriuretic peptide and *β*-myosin heavy chain. Cardiac hypertrophy is a distinguishing feature of some cardiovascular diseases. Our previous study showed that sodium ferulate (SF) alleviates myocardial hypertrophy induced by coarctation of the abdominal aorta, and these protective effects may be related to the inhibition of protein kinase C (PKC) and mitogen-activated protein kinase (MAPK) signaling pathways. This study investigated the inhibitory effect and mechanism of SF on myocardial hypertrophy in spontaneously hypertensive rats (SHRs). The effects of SF on cardiac hypertrophy were evaluated using echocardiographic measurement, pathological analysis, and detection of atrial natriuretic peptide (ANP) and *β*-myosin heavy chain (*β*-MHC) expression. To investigate the mechanisms underlying the anti-hypertrophic effects of SF, the calcium-sensing receptor (CaSR), calcineurin (CaN), nuclear factor of activated T cells 3 (NFAT3), zinc finger transcription factor 4 (GATA4), protein kinase C beta (PKC-*β*), Raf-1, extracellular signal-regulated kinase 1/2 (ERK 1/2), and mitogen-activated protein kinase phosphatase-1 (MKP-1) were detected by molecular biology techniques. Treatment with SF ameliorated myocardial hypertrophy in 26-week-old SHRs. In addition, it downregulated the levels of ANP, *β*-MHC, CaSR, CaN, NFAT3, phosphorylated GATA4 (p-GATA4), PKC-*β*, Raf-1, and p-ERK 1/2; and upregulated the levels of p-NFAT3 and MKP-1. These results suggest that the effects of SF on cardiac hypertrophy are related to regulation of the CaSR-mediated signaling pathway.

## Introduction

Left ventricular hypertrophy is the common complication of hypertension and is an important cause of death in patients with cardiovascular disease (Zhang et al., 2012). As an adaptive response of cardiomyocytes to external or internal stimuli, myocardial hypertrophy is characterized by increased protein synthesis and enlargement of cardiomyocytes, and the reactivation of fetal genes such as atrial natriuretic peptide (ANP) and *β*-myosin heavy chain (*β*-MHC) (Kang and Hu, 2007; Roger et al., 2011; Grossman and Paulus, 2013). Although initial cardiac hypertrophy is a compensatory mechanism to increase cardiac output, prolonged or excessive cardiac hypertrophy leads to decompensated dilated cardiomyopathy, cardiac dysfunction, arrhythmia, fibrotic disease, and even sudden death (Devereux and Roman, 1999; Santulli and Iaccarino, 2016). Unfortunately, because the mechanism of cardiac hypertrophy is complex, there is currently no optimal therapy for this condition. Therefore, it is urgent to identify novel therapeutic methods or drugs to treat myocardial hypertrophy.

The extracellular calcium-sensing receptor (CaSR) is a member of the G-protein-coupled receptor C family and is expressed at multiple sites including the parathyroid, thyroid, kidney, bone, and gastrointestinal tract (Brown et al., 1993; Zaidi et al., 1993; McGehee et al., 1997; Dufner et al., 2005; Theman and Collins, 2009; Riccardi and Brown, 2010). CaSR in cardiomyocytes was first discovered in 2003 (Wang et al., 2003). It plays a crucial role in cell proliferation and differentiation, apoptosis, hormone secretion, and calcium homeostasis *in vivo* (Giudice et al., 2019), and is expressed in the cardiac tissue of rats. The overexpression of CaSR contributes to the development of cardiac hypertrophy (Wang et al., 2008; Liu et al., 2016). Therefore, CaSR may be a promising therapeutic target for the treatment of myocardial hypertrophy.

Sodium ferulate (SF) is derived from ferulate acid extracted from Chinese herbal medicines (*Radix Angelicae Sinensis* and *Lignsticum chuangxiong*), and can be synthesized manually. SF is 3-methoxy-4-hydroxy-cinnamate sodium with multiple pharmacological effects such as anti-platelet, anti-oxidation, free radical scavenging, anti-inflammatory, and anti-thrombotic properties (Wei et al., 2016). For several decades, SF has been widely used in China to treat cardiovascular and cerebrovascular diseases and to prevent thrombosis (Wang and Ou-Yang, 2005). Our previous study showed that SF alleviates myocardial hypertrophy induced by coarctation of the abdominal aorta (Luo et al., 2019). In this study, we evaluated the pharmacological effects of SF on cardiac remodeling in spontaneously hypertensive rats (SHRs) and the underlying mechanisms, focusing on the signaling pathway mediated by CaSR.

## Materials and Methods

### Animals and Experimental Design

A total of 40 male SHRs and 10 male Wistar Kyoto (WKY) rats, 13 weeks old, and specific pathogen-free were provided by the Beijing Vital River Laboratory Animal Technology Co., Ltd. (certificate number: SCXK 2016-006; Beijing, China). To investigate the effects of SF, at 1 week after acclimatization, the SHRs were administered SF at 20 mg/kg/day (SF-20), 40 mg/kg/day (SF-40), or 80 mg/kg/day (SF-80); SHRs and age-matched male WKY rats as normal controls were given distilled water. The dosage of SF and the course of treatment were determined by clinical instruction and our preliminary experiment. Animals were intragastrically administered SF between 9:00 and 11:00 for 12 consecutive weeks. All animals were housed in specific pathogen-free animal facilities at Zunyi Medical University (Guizhou, China) with controlled light cycles (12 h light/12 h dark); food and drinking water were available *ad libitum*. All studies were approved by the animal care and use committee of Zunyi Medical University.

### Blood Pressure Measurements

The tail artery blood pressure of eight conscious rats was randomly monitored in each group weekly using the CODA Non-Invasive Blood Pressure System (Kent Scientific Corp., Torrington, CT, United States) once a week in a quiet, warm, and dark environment.

### Left Ventricular Ultrasonography in Rats

Left ventricular structure and function index of the rats were measured with the Vevo2100 High Resolution Animal Ultrasound Imaging System (Visual Sonics Corporation, Toronto, Canada) under anesthesia with pentobarbital sodium (30 mg/kg). The parameters included diastole of the left ventricular anterior wall (LVAW; d) thickness, diastole of the left ventricular posterior wall (LVPW; d) thickness, diastole of the left ventricular internal diameter (LVID; d), and average cardiac stroke volume (SV). To observe whether myocardial hypertrophy had been formed in 14-week-old SHRs, we randomly selected 4 rats in SHRs and WKY rats for cardiac ultrasound testing. After 12 weeks of SF administration, six SHRs and WKY rats were randomly selected in each group for cardiac ultrasound testing to observe the effect of SF.

### Analysis of Myocardial Hypertrophy Parameters

After 12 weeks of SF administration, the hearts of all rats were immediately removed under the anesthesia state and rinsed with cold phosphate-buffered saline. The left ventricular wall (including interventricular septum) and right ventricular wall were isolated and weighed after being blotted dry. As left ventricular hypertrophic parameters, the left ventricular hypertrophy index (LVHI, ratio of the left ventricle and body weight) and ratio of the left ventricular weight/right ventricular weight (LVW/RVW) were calculated.

### Histologic Analysis

To observe the pathohistology of the myocardial samples, five rats were randomly selected in each group for Histologic analysis. The heart specimens were routinely fixed in 4% formaldehyde solution for 48 h and embedded in paraffin. Sections (3.5 μm thick) underwent hematoxylin and eosin (H&E) staining and Modified Masson’s trichrome staining for morphological analysis using the BX 43 Olympus Image Analysis System (Olympus, Tokyo, Japan) (Sun et al., 2018). The degree of hypertrophy of myocardial cells can be observed in transverse sections. The cross-sectional area of the cardiomyocytes was calculated using Image Pro-Plus 6.0 software, ensuring that there were at least 60 cardiomyocytes per left ventricle section. Interstitial and perivascular fibrosis was quantified by calculating the percentage area of collagen staining. To observe the effect of SF on the left ventricular myocardial ultrastructure in SHRs, the rat heart tissue specimens (1 mm^3^) were randomly taken in each group to fix in 2.5% cold glutaraldehyde, washed in 0.1 mol/L phosphate buffer three times, fixed in 1% osmium tetroxide, dehydrated in an ethanol series, embedded in epoxy propane, polymerized in an oven at 70°C, sectioned into 70 nm slices, and finally examined under a transmission electron microscope (JEM-1230; JEOL Co., Ltd., Tokyo, Japan).

### Immunohistochemistry Analysis

To observe the protein levels of ANP and *β*-MHC in the cardiac tissue, five rats were randomly selected in each group for Immunohistochemistry analysis. The paraffin-embedded sections (3.5 µm) were replaced in citrate buffer (pH 6.0) and boiled in a microwave on full power for antigen retrieval after deparaffinization. Thereafter, the slices were blocked in goat serum (Zsbio Commerce Store, Beijing, China) for 30 min and then incubated separately with anti-ANP antibody (1:200; Santa Cruz Biotechnology, Dallas, TX, United States) and anti-*β*-MHC antibody (1:200; Santa Cruz) at 4°C for 17 h. Subsequently, the sections were incubated with horseradish peroxidase-conjugated goat and anti-rabbit secondary antibodies for 20 min at 37°C, followed by visualization with a DAB Kit (Zsbio Commerce Store). The Olympus Image Analysis System (Olympus) was used for morphological analysis.

### Gene Expression Analysis

Five rats were randomly selected in each group for Gene expression analysis. Total RNA was extracted from cardiac tissues using TRIzol™ reagent (MRC Co., Cincinnati, OH, United States) and the Reverse Transcriptase System (TaKaRa Biotechnology Co., Ltd., Dalian, China) by the C1000 Touch™ Thermal Cycler (Bio-Rad, Hercules, CA, United States). Quantitative PCR (qPCR) was conducted with the iQ^TM^SYBR® Green Supermix (Bio-Rad) using the iCycler iQ Real-Time PCR Detection System (Bio-Rad). The amplification specificity was checked by the melting curve following the manufacturer’s instructions. The internal control gene was GAPDH. The primers were designed and synthesized by Sangon Biotech Co., Ltd. (Shanghai, China), and are shown in Table 1. The target gene (threshold cycle values, Ct values) was normalized to GAPDH of the same sample.

**TABLE 1:** Sequences of the primers pairs used for real-time PCR.

Gene name	GenBank accession no	Primer sequence (5′- 3′)
ANP	NM_012612	F: GGG​GGT​AGG​ATT​GAC​AGG​AT
		R: CTC​CAG​GAG​GGT​ATT​CAC​CA
β-MHC	NM_017240	F: TGG​CAC​CGT​GGA​CTA​CAA​TA
		R: TAC​AGG​TGC​ATC​AGC​TCC​AG
CaSR	NM_001309638.1	F: CTT​TGT​GCT​GGG​TGT​CTT​CA
		R: AAC​AAG​GAG​CTG​GAG​AAG​CA
CaN	NM_017041	F: GCA​GGC​TGG​AAG​AAA​GTG​TC
		R: AAG​GCC​CAC​AAA​TAC​AGC​AC
NFAT_3_	NM_001107264	F: TCT​TCA​GGA​CCT​CTG​CCC​TA
		R: AGC​CTA​GGA​GCT​TGA​CCA​CA
GATA_4_	NM_144730	F: TCT​CAC​TAT​GGG​CAC​AGC​AG
		R: CGA​GCA​GGA​ATT​TGA​AGA​GG
PKC-β	NM_012713.3	F: AAG​ACA​TTC​TGT​GGC​ACT​CCA​GAC
		R: AGC​CAA​CAT​TTC​ATA​CAG​CAG​GAC
Raf-1	NM_012639	F: CTT​GCA​CGA​CTG​CCT​TAT​GA
		R: TGA​GTG​GAA​CGT​GAT​CCA​AA
ERK 1/2	NM 053842	F: GTT​CCC​AAA​CGC​TGA​CTC​CAA
		R: GTA​AGT​CGT​CCA​GCT​CCA​TGT​CAA
MKP-1	NM_053769	F: TGA​AGC​AGA​GGC​GGA​GTA​TT
		R: TGA​TGG​GGC​TTT​GAA​GGT​AG
GAPDH	NM_017008	F: AGA​CAG​CCG​CAT​CTT​CTT​GT
		R: CTT​GCC​GTG​GGT​AGA​GTC​AT

### Western Blot Analysis

Three rats were randomly selected in each group for Western blot analysis. The total protein of cardiac tissue samples was extracted using radioimmunoprecipitation assay (RIPA) lysis buffer supplemented with phenylmethyl sulfonylfluoride (Beyotime, Shanghai, China). The protein concentrations were detected by the BCA Protein Assay Kit (Beyotime, Shanghai, China). The protein samples were resolved on 6–12% gradient sodium dodecyl sulfate-polyacrylamide gel electrophoresis by the Mini-Protean^®^ Tetra system (Bio-Rad), and transferred to polyvinylidene fluoride (0.45 μm) membranes (Millipore Trading, MT, United States) by Trans-Blot^®^ Turbo™ Transfer system (Bio-Rad). The membranes were blocked in 5% defatted milk, followed by incubation with the following primary antibodies: anti-ANP polyclonal antibody (1: 500; Santa Cruz), anti-*β*-MHC monoclonal antibody (1:500; Santa Cruz), anti-phosphorylated nuclear factor of activated T cells 3 (p-NFATc3) monoclonal antibody (1:500; Santa Cruz), anti-CaSR polyclonal antibody (1:1,000; Proteintech Group, Rosemont, IL, United States), anti-extracellular signal-regulated kinase 1/2 (ERK1/2) polyclonal antibody (1:2000; Proteintech Group), anti-*β*-actin monoclonal antibody (1:20,000; Proteintech Group), anti-lamin B1 polyclonal antibody (1:3000; Proteintech Group), anti-GAPDH polyclonal antibody (1:20,000; Proteintech Group), anti-NFATc3 monoclonal antibody (1:1,000; Santa Cruz), anti-p-ERK1/2 monoclonal antibody (1:2000; Cell Signaling Technology, United States), anti-calcineurin (CaN) polyclonal antibody (1:3000; Abcam, Cambridge, MA, United States), anti-zinc finger transcription factor 4 (GATA4) polyclonal antibody (1:1,000; Abcam), anti-p-GATA4 polyclonal antibody (1:1,000; Abcam), anti-protein kinase C beta (PKC-*β*) monoclonal antibody (1:1,000; Abcam), anti-Raf-1 polyclonal antibody (1:1,000; Abcam), and anti-mitogen-activated protein kinase phosphatase-1 (MKP-1) polyclonal antibody (1:500; Abcam). In some experiments, nuclear proteins were isolated from heart samples with the Nucleoprotein Extraction Kit (Solarbio Co., Ltd., Beijing, China). Samples were incubated with secondary antibodies (1:5000 dilution); GAPDH or lamin B1 (served for nuclear protein control) was used to normalize protein loading. Immunoassay and analysis were carried out using the Gel Imaging Analysis System with Quantity One 1-D Analysis Software v4.52 (Bio-Rad).

### Statistical Analysis

All statistical analyses were performed using SPSS 18.0 software (SPSS Inc., Chicago, IL, United States), and data are presented as the mean ± standard error of the mean (SEM). *p* < 0.05 were considered statistically significant. The differences between groups were analyzed by one-way analysis of variance. If the variance was homogeneous, the least significant difference method was used; if the variance was heterogeneous, Dunnett’s T3 test was used.

## Results

### Effects of Sodium Ferulate on Blood Pressure Regulation in Spontaneously Hypertensive Rats

**FIGURE 1:**
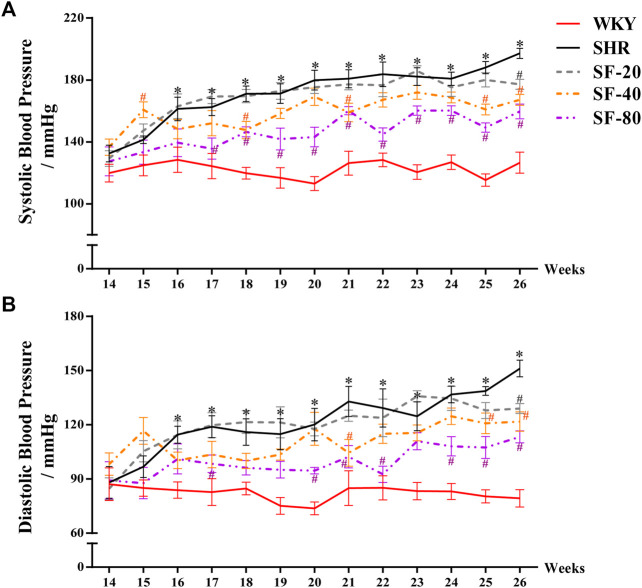
Effect of SF on blood pressure in SHRs. SF-20, SF-40, and SF-80 were administered to SHRs for 12 weeks; SHRs and age-matched male WKY rats were given distilled water as model (SHRs) and normal (WKY) controls. **(A)** Effect of SF on systolic blood pressure in SHRs (*n* = 8). **(B)** effect of SF on diastolic blood pressure in SHRs (*n* = 8). Data are presented as mean ± SEM. ^*^significantly different from WKY rats at *p* < 0.05; ^#^significantly different from SHRs at *p* < 0.05.

SHRs are a typical animal model of cardiac hypertrophy and hypertension. Their blood pressure increases gradually with age. To observe the effect of SF on blood pressure in SHRs, the tail artery blood pressure of conscious rats was monitored once a week. As shown in Figure 1, the systolic blood pressure of WKY rats was basically stable within 122.4 ± 1.4 mmHg, and the diastolic blood pressure was stable within 82.2 ± 1.1 mmHg from 14 to 26 weeks. The systolic blood pressure of 26-week-old SHRs reached 197.2 ± 3.3 mmHg and the diastolic blood pressure reached 151.1 ± 4.6, which were significantly higher than that of WKY rats (*p* < 0.05). However, SF caused the blood pressure of SHRs to decrease, especially at 80 mg/kg (*p* < 0.05).

### Effects of Sodium Ferulate on Left Ventricular Structure and Function Index in Spontaneously Hypertensive Rats

**FIGURE 2:**
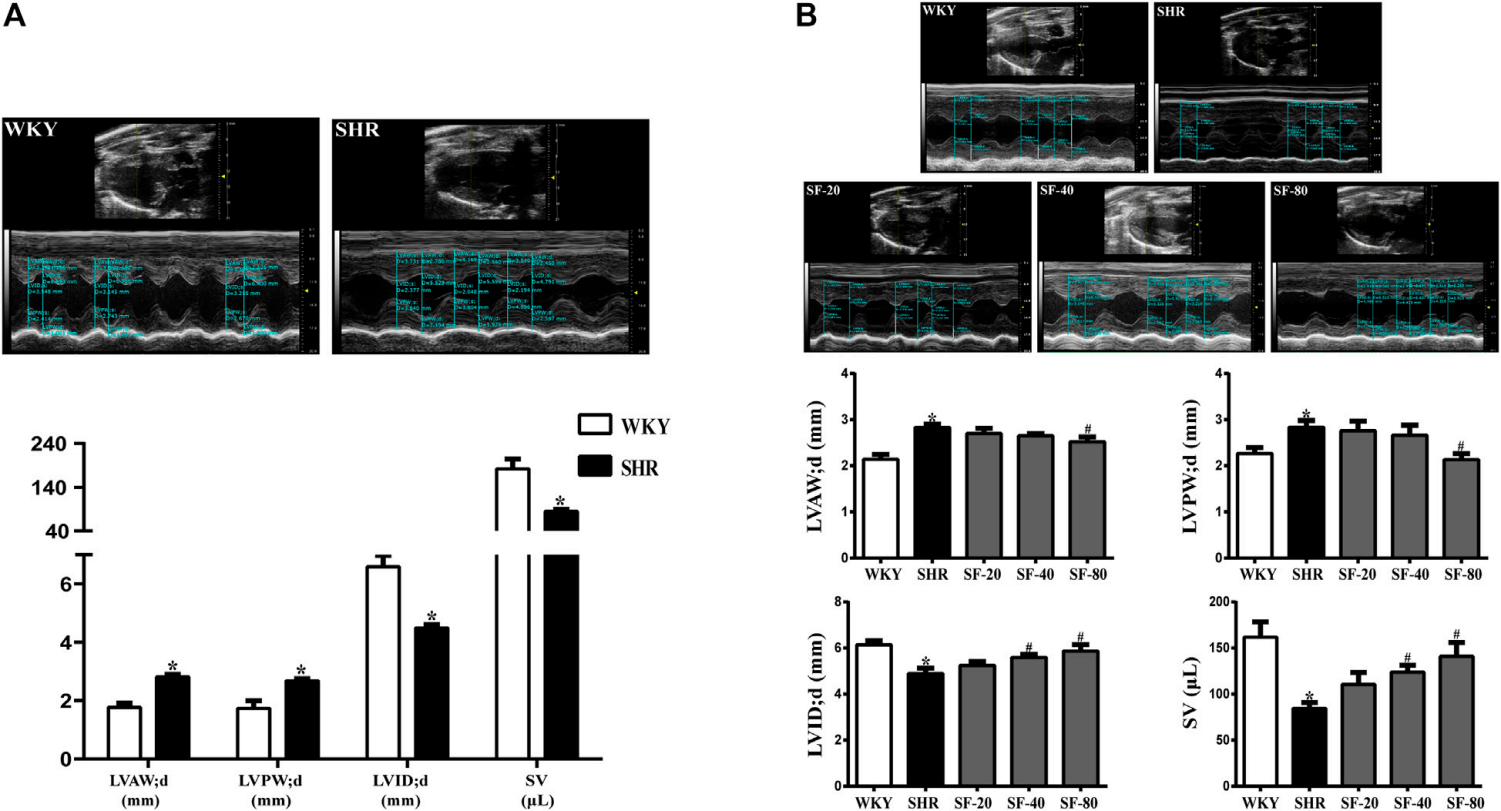
Detection of left ventricular structure and function index in SHRs by small animal ultrasound. **(A)** The ventricular wall thickness and cardiac function of 14-week-old SHRs were significantly abnormal compared to WKY rats. (*n* = 4). **(B)** SF-40 and SF-80 significantly improved left ventricular structure and function in 26-week-old SHRs. (*n* = 6). Data are presented as mean ± SEM. ^*^significantly different from WKY rats at *p* < 0.05; ^#^significantly different from SHRs at *p* < 0.05.

As shown in Figure 2A, the LVAW; d and LVPW; d increased, but the LVID; d and SV significantly decreased in 14-week-old SHRs compared to WKY rats of the same age (*p* < 0.05). After 12 weeks of administration, the left ventricular function was detected by ultrasound. Compared with WKY rats, LVAW; d and LVPW; d were increased and LVID; d and SV were reduced in 26-week-old SHRs (Figuer 2B). SF-40 and SF-80 significantly improved left ventricular structure and function in SHRs at 26 weeks of age (*p* < 0.05).

### Effects of Sodium Ferulate on the Left Ventricular Index in Spontaneously Hypertensive Rats

**FIGURE 3:**
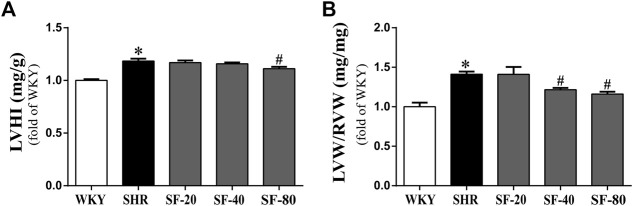
SF can decrease the left ventricular hypertrophy index in SHRs. **(A)** Ratio of LVHI. (*n* = 10). **(B)** ratio of LVW/RVW. (*n* = 10). Data are presented as mean ± SEM. ^*^significantly different from WKY at *p* < 0.05; ^#^significantly different from SHRs at *p* < 0.05.

Pressure overload leading to myocardial hypertrophy usually manifests as hypertrophy of the left ventricle—mainly increases in left ventricular weight. Thus, we evaluated the left ventricular weight of rats, and the results are shown in Figure 3. The LVHI and LVW/RVW were significantly increased in SHRs (*p* < 0.05). SF-40 and SF-80 significantly decreased the LVHI and LVW/RVW in SHRs (*p* < 0.05).

### Effects of Sodium Ferulate on Left Ventricular Histomorphology in Spontaneously Hypertensive Rats

**FIGURE 4:**
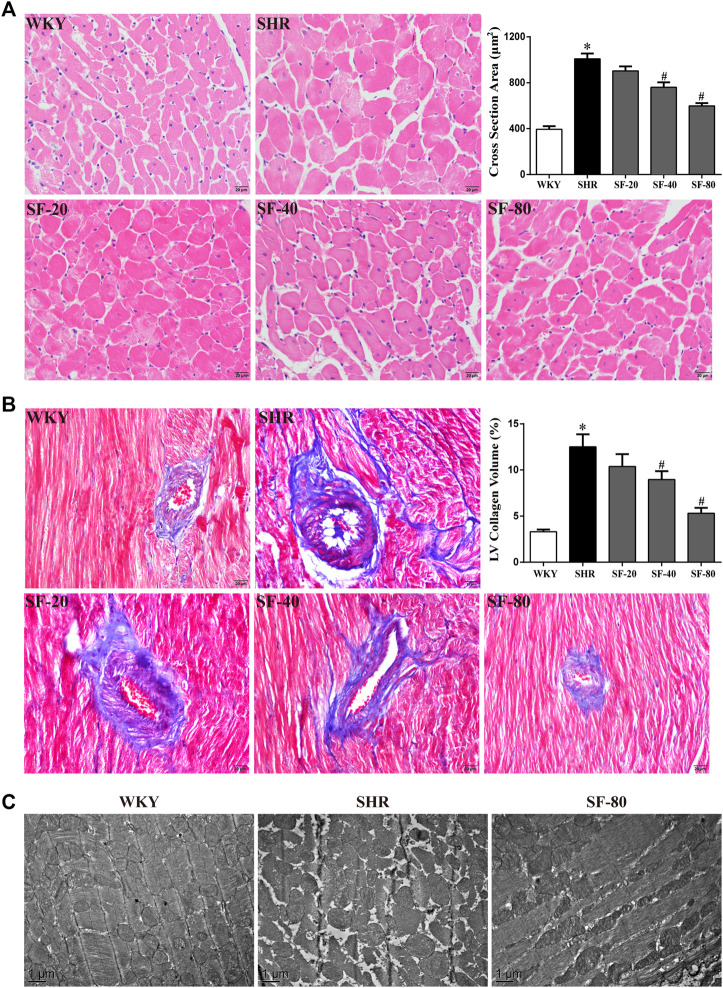
SF can improve left ventricular histomorphology in SHRs. **(A)** Representative photomicrographs of transection of myocardial tissue in rats (scale bar = 20 μm). (*n* = 5). **(B)** representative photomicrographs of collagen deposition of myocardial tissue in rats (scale bar = 20 μm). (*n* = 5). **(C)** representative photographs of ultrastructure of myocardial tissue in rats (bar = 1 μm). SF significantly inhibited the increase of cardiomyocytes and the deposition of collagen fibers and improved the ultrastructure of cardiomyocytes. Data are presented as mean ± SEM. ^*^significantly different from WKY at *p* < 0.05; ^#^significantly different from SHRs at *p* < 0.05.

To further observe the effect of SF on the pathomorphology of left ventricular myocardium in rats, we performed H&E and Masson’s trichrome staining. As shown in Figure 4A (H&E staining) and Figure 4B (Masson’s trichrome staining), compared with WKY rats, the cross-sectional area of cardiomyocytes was significantly increased in SHRs (*p* < 0.05). Large amounts of blue-stained collagen fibers were deposited in the perivascular and myocardial interstitium of SHRs (*p* < 0.05). Transmission electron microscopy showed that the myofilaments of WKY rats were arranged in an orderly manner, the sarcomere band was clear, the mitochondria were abundant, and the Z-line was normal. However, myofilaments were dissolved, mitochondria were swollen and vacuolated, the tissue gap was large, and the Z-lines were fractured in SHRs (Figure 4C). SF, especially 80 mg/kg, significantly improved the histomorphological damage.

### Effects of Sodium Ferulate on Atrial Natriuretic Peptide and *β*-Myosin Heavy Chain Levels in Spontaneously Hypertensive Rats

**FIGURE 5:**
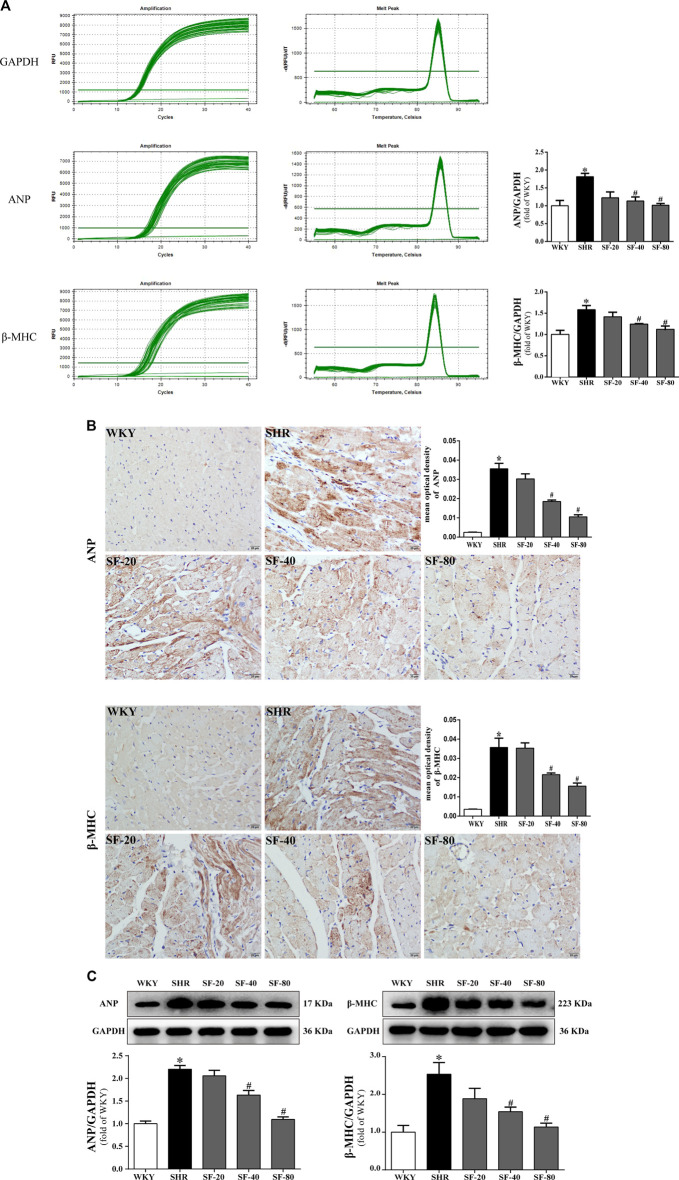
SF downregulates ANP and *β*-MHC in the myocardial tissue of SHRs. **(A)** Quantitative analysis of ANP and *β*-MHC mRNA levels. (*n* = 5). **(B)** immunohistochemical staining of ANP and *β*-MHC (bar = 20 μm). (*n* = 5). **(C)** quantitative analysis of ANP and *β*-MHC protein levels. (*n* = 3). ANP and *β*-MHC mRNA and protein level were significantly higher in the myocardial tissue of SHRs than WKY rats. SF significantly downregulated the levels of ANP and *β*-MHC in the myocardial tissue of SHRs. Data are presented as mean ± SEM. ^*^significantly different from WKY at *p* < 0.05; ^#^significantly different from SHRs at *p* < 0.05.

As markers of myocardial hypertrophy, ANP and *β*-MHC in the rat myocardium were detected at the transcriptional and translational levels by qPCR, and immunohistochemical staining and western blotting, respectively. ANP and *β*-MHC mRNA levels were significantly increased in SHRs (Figure 5A). Immunohistochemical staining showed that ANP and *β*-MHC proteins were mainly expressed in the cytoplasm of the myocardium with brown staining, and the expression of ANP and *β*-MHC proteins in the myocardium of 26-week-old SHRs was significantly higher than that in WKY rats (*p* < 0.05; Figure 5B). The quantitative Western blot results were consistent with the qPCR results (Figure 5C). Significant downregulation of ANP and *β*-MHC in the myocardial tissue of SHRs was caused by SF, especially 80 mg/kg (*p* < 0.05).

### Effects of Sodium Ferulate on Calcium-Sensing Receptor, Calcineurin, Nuclear Factor of Activated T cells 3, and Zinc Finger Transcription Factor Levels in Spontaneously Hypertensive Rats

**FIGURE 6:**
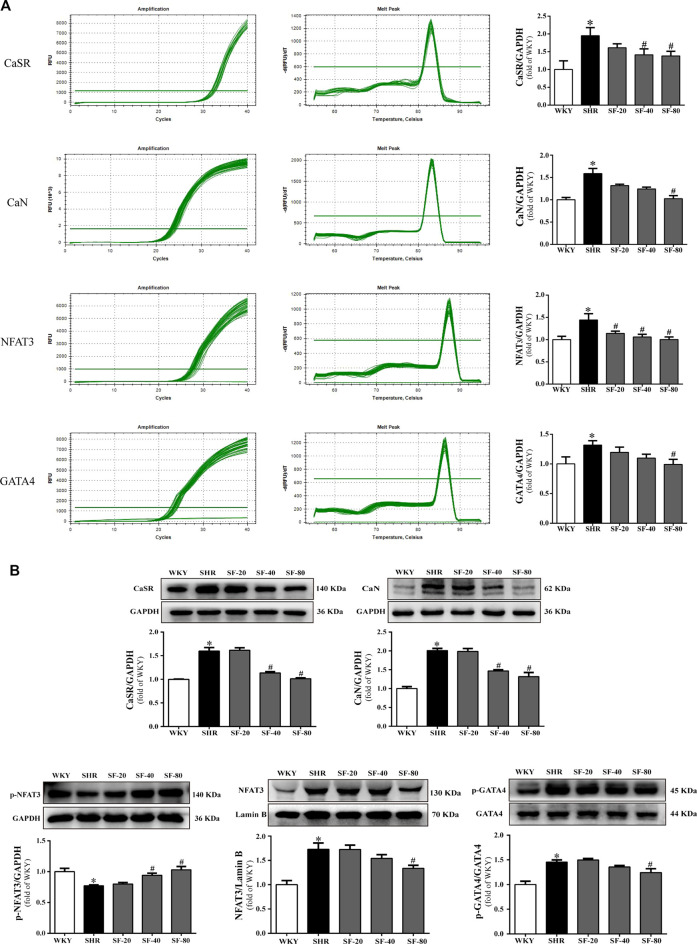
SF improves cardiac hypertrophy by regulating CaSR, CaN, NFAT3, and GATA4 levels. **(A)** Quantitative analysis of CaSR, CaN, NFAT3, and GATA4 mRNA levels. (*n* = 5). **(B)** quantitative analysis of CaSR, CaN, p-NFAT3, NFAT3, and GATA4 protein level. (*n* = 3). The levels of CaSR, CaN, NFAT3, and GATA4 were significantly upregulated in the myocardium of SHRs at the transcriptional and translational levels, and the level of p-NFAT3 protein in the cytoplasm was significantly downregulated. SF downregulated the levels of CaSR, CaN, NFAT3, and GATA4 and upregulated the level of p-NFAT3 protein in the cytoplasm in the left ventricular myocardial tissue of SHRs. Data are presented as mean ± SEM. ^*^significantly different from WKY at *p* < 0.05; ^#^significantly different from SHRs at *p* < 0.05.

As shown in Figure 6A and Figure 6B, the levels of CaSR, CaN, NFAT3, and GATA4 were significantly upregulated in the myocardium of SHRs at the transcriptional and translational levels (*p* < 0.05). At the same time, the level of *p*-NFAT3 protein in the cytoplasm was significantly downregulated in the myocardium of SHRs (*p* < 0.05). SF, especially 80 mg/kg, downregulated the levels of CaSR, CaN, NFAT3, and GATA4 in the left ventricular myocardial tissue of 26-week-old SHRs and upregulated the level of p-NFAT3 protein in the cytoplasm (*p* < 0.05).

### Effects of Sodium Ferulate on Protein kinase C beta, Raf-1, Extracellular signal-regulated kinase 1/2, and Mitogen-activated protein kinase phosphatase-1 Levels in Spontaneously Hypertensive Rats

**FIGURE 7:**
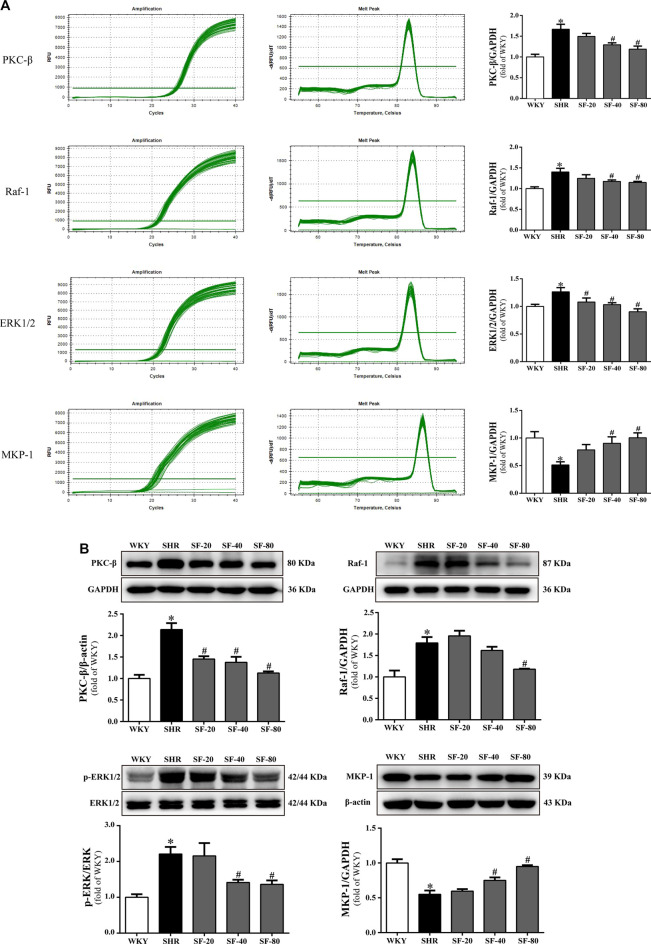
SF improves cardiac hypertrophy by regulating PKC-*β*, Raf-1, ERK1/2, and MKP-1 levels. **(A)** Quantitative analysis of PKC-*β*, Raf-1, ERK1/2, and MKP-1 mRNA levels. (*n* = 5). **(B)** quantitative analysis of PKC-*β*, Raf-1, ERK1/2, and MKP-1 protein levels. (*n* = 3). The levels of PKC-*β*, Raf-1, and ERK1/2 were significantly upregulated and the level of MKP-1 was significantly downregulated in the myocardium of SHRs at the transcriptional and translational levels. SF significantly downregulated the levels of PKC-*β*, Raf-1, ERK1/2, and significantly upregulated the level of MKP-1 in the left ventricular myocardial tissue of SHRs. Data are presented as mean ± SEM. ^*^significantly different from WKY at *p* < 0.05; ^#^significantly different from SHRs at *p* < 0.05.

The levels of PKC-β, Raf-1, and ERK1/2 are significantly upregulated and MKP-1 expression was significantly downregulated in the myocardium of SHRs at the transcriptional and translational levels (*p* < 0.05). SF, especially 80 mg/kg, significantly downregulated the levels of PKC-β, Raf-1, ERK1/2 and significantly upregulated the expression of MKP-1 in the left ventricular myocardial tissue of 26-week-old SHRs (*p* < 0.05; Figure 7A and Figure 7B).

## Discussion

Myocardial hypertrophy is a risk factor for human health and an independent risk factor for increased cardiovascular mortality (Smith and Squiers, 2013; Oka et al., 2014). As an ideal model for studying cardiac hypertrophy, the genetic background of hypertension in SHRs is consistent with the course of hypertension in humans (Damatto et al., 2016). Because WKY rats have the same genetic background as SHRs, they are often used as normal control rats. In this study, we used SHRs to observe the inhibitory effect of SF on myocardial hypertrophy. The results showed that SF slightly decreased blood pressure, improved cardiac function and decreased the hypertrophy parameters of the left ventricle, ameliorated the histological features of cardiac hypertrophy, and downregulated the levels of ANP and *β*-MHC in SHRs. These findings clearly demonstrate that SF can alleviate myocardial hypertrophy in SHRs.

The blood pressure of SHRs increases continuously with age. Compared with age-matched WKY rats, the left ventricular wall thickness was significantly increased and the left ventricular diameter was significantly decreased in 14-week-old SHRs, indicative of the development of left ventricular hypertrophy. The left ventricular wall thickness increased, and the left ventricular diameter and SV decreased with the increase in age to 26-week-old SHRs, and LVHI and LVW/RVW, and the cross-sectional area of cardiomyocytes were significantly increased. These findings suggested that the left heart structure and function of 26-week-old SHRs were abnormal. Pathological cardiac hypertrophy is often accompanied by significant myocardial fibrosis (Jiang et al., 2014). In this study, there was a large amount of collagen fiber deposition in the myocardial stroma and perivascular area of SHRs, suggesting that long-term stress overload not only causes cardiac hypertrophy but also leads to myocardial fibrosis. Myocardial mitochondria are an important part of myocardial energy production. Mitochondrial swelling reflects the dysfunction of sodium and potassium ATP enzymes, and the integrity of myofilament structure is the structural basis to ensure the normal systolic and diastolic function of myocardium (Wang et al., 2019). Transmission electron microscopy showed that the structure of mitochondria and myofilaments in SHRs had obvious lesions. As markers of myocardial hypertrophy, the levels of ANP and *β*-MHC were significantly upregulated in the myocardial tissue of SHRs. These results provide direct and indirect evidence of left ventricular hypertrophy caused by a continuous increase in blood pressure. As an antagonist of the endothelin receptor, SF has certain antihypertensive effects on SHRs. Meanwhile, SF significantly reduced the left ventricular wall thickness and left ventricular hypertrophy index, increased the left ventricular diameter and SV, improved myocardial pathological morphology and ultrastructure, and downregulated the levels of ANP and *β*-MHC in SHRs. Thus, SF inhibited the process of myocardial hypertrophy in SHRs, in part through its hypotensive effect. This is consistent with previous observations on the effect of SF on myocardial hypertrophy induced by abdominal aortic coarctation in rats.

**FIGURE 8:**
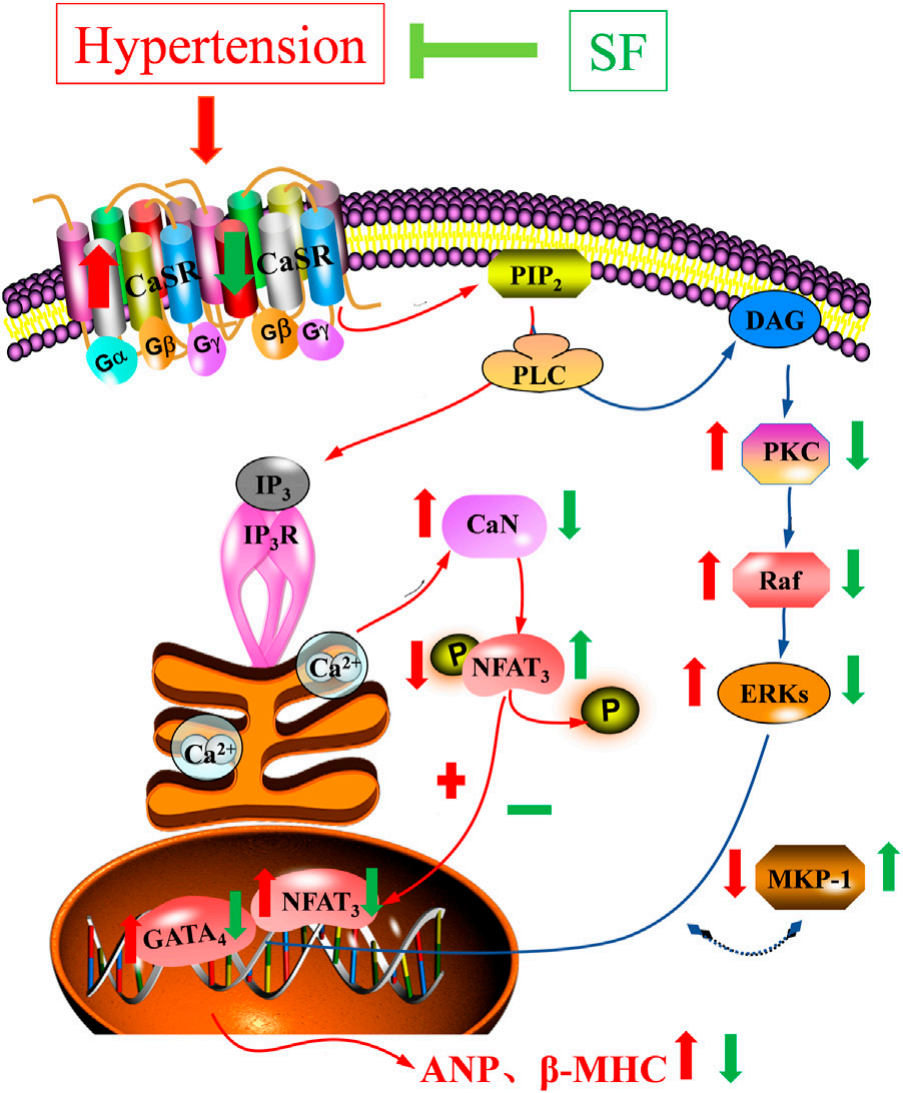
Schematic diagram of the mechanism underlying inhibition of cardiac hypertrophy by SF in SHRs.

The CaSR plays an important role in mediating pathological myocardial hypertrophy and in cardiovascular diseases such as myocardial ischemia-reperfusion injury, heart failure, and vascular calcification (Zhang et al., 2014; Paccou et al., 2014; Toka and Pollak, 2014). CaSR is a dimeric family C G protein-coupled receptor, and is activated by calcium ions. Activated CaSR can induce the activation of phospholipase C. Hydrolysis of phosphatidylinositol diphosphate on the plasma membrane to second messengers, triphosphate inositol (IP3) and diacylglycerol (DAG), stimulates intracellular signal transduction (Kifor et al., 1997). IP3 can cause the release of calcium from the sarcoplasmic reticulum into the cytoplasm to further activate CaN, which initiates signaling to the nucleus (Zhao et al., 2015). Activated CaN facilitates binding to its primary downstream effector, NFAT3 (Kehat and Molkentin, 2010). Normally, NFAT3 is hyperphosphorylated and chelated in the cytoplasm, followed by rapid translocation to the nucleus after calcineurin-mediated dephosphorylation, binding and activation of *p*-GATA4 (Wilkins et al., 2004). The *p*-GATA4 can initiate the expression of hypertrophy response genes such as ANP and *β*-MHC, which participate in the development of myocardial hypertrophy (Liu et al., 2015). DAG can specifically activate PKC with calcium involvement (Kifor et al., 2001; Allo et al., 1992). The activation of PKC-*β* and its downstream signaling pathways play a regulatory role in the development of cardiac hypertrophy and heart failure (Takeishi et al., 1998; Takahashi et al., 2005). Moreover, the activation of PKC-*β* can activate Raf-1 and the MAPK signaling cascade in the cytoplasm, which in turn mediates cardiac hypertrophy (Axmann et al., 1998). ERK1/2 is an important member of the MAPK family, and its phosphorylation can lead to interaction with GATA4 and myocardial hypertrophy (Hu et al., 2017). ERK1/2 can be inactivated by MKP-1, which inhibits the PKC/MAPK signaling pathway and delays the process of myocardial hypertrophy (Koh, 2015). In this study, SF downregulated the levels of CaST, CaN, NFAT3, p-GATA4, PKC-*β*, Raf-1, p-ERK1/2, and upregulated the levels of p-NFAT3 and MKP-1 in the myocardium of SHRs. The results showed that the CaSR-mediated and PKC-MAPK signaling pathways were involved in the anti-hypertrophic effects of SF (Figure 8). These findings are consistent with our previous experimental results in a rat model of myocardial hypertrophy induced by abdominal aortic coarctation (Luo et al., 2019). In a follow-up study, we will design cellular experiments to determine the specific mechanism of SF, used the inhibitors of CaSR-mediated signaling pathway and the inhibitors of PKC-MAPK signaling pathway. This provide more sufficient evidence to prove that SF influence on cardiac hypertrophy via CaSR singnaling pathway.

In summary, combined with our previous research, these results strongly demonstrate that SF can inhibit cardiac hypertrophy in rats through regulating the CaSR-mediated signal pathway and inhibiting the PKC-MAPK signaling pathways.

## Data Availability

The raw data supporting the conclusion of this article will be made available by the authors, without undue reservation, to any qualified researcher.
