# Supplementary material for: Effects of Sodium Ferulate on Cardiac Hypertrophy Are via the CaSR-Mediated Signaling Pathway
Source: Front Pharmacol. 2021 Oct 6;12:674570. doi: 10.3389/fphar.2021.674570 (PMC8526863; doi:10.3389/fphar.2021.674570)
Supplement: Supplementary file 1 [file Table1.DOCX]

| **Gene name** | **GenBank accession No.** | **Primer sequence (5’- 3’)** |
| --- | --- | --- |
| ANP | NM_012612 | F: GGGGGTAGGATTGACAGGAT  R: CTCCAGGAGGGTATTCACCA |
| β-MHC | NM_017240 | F: TGGCACCGTGGACTACAATA  R: TACAGGTGCATCAGCTCCAG |
| CaSR | NM_001309638.1 | F: CTTTGTGCTGGGTGTCTTCA  R: AACAAGGAGCTGGAGAAGCA |
| CaN | NM_017041 | F: GCAGGCTGGAAGAAAGTGTC  R: AAGGCCCACAAATACAGCAC |
| NFAT_3_ | NM_001107264 | F: TCTTCAGGACCTCTGCCCTA  R: AGCCTAGGAGCTTGACCACA |
| GATA_4_ | NM_144730 | F: TCTCACTATGGGCACAGCAG  R: CGAGCAGGAATTTGAAGAGG |
| PKC-β | NM_012713.3 | F: AAGACATTCTGTGGCACTCCAGAC  R: AGCCAACATTTCATACAGCAGGAC |
| Raf-1 | NM_012639 | F: CTTGCACGACTGCCTTATGA  R: TGAGTGGAACGTGATCCAAA |
| ERK 1/2 | NM 053842 | F: GTTCCCAAACGCTGACTCCAA  R: GTAAGTCGTCCAGCTCCATGTCAA |
| MKP-1 | NM_053769 | F: TGAAGCAGAGGCGGAGTATT  R: TGATGGGGCTTTGAAGGTAG |
| GAPDH | NM_017008 | F: AGACAGCCGCATCTTCTTGT  R: CTTGCCGTGGGTAGAGTCAT |
